# Supplementary material for: Investigation on the influence of unbalanced shaft component in gearbox on displacement using the Newmark-β method
Source: PLoS One. 2025 Nov 5;20(11):e0335632. doi: 10.1371/journal.pone.0335632 (PMC12588525; doi:10.1371/journal.pone.0335632)
Supplement: Attachment — Submitted filename: Reviewers comments.docx [file pone.0335632.s002.docx]

Effect of unbalanced parameters on power transmission shaft displacement in gearbox system using the Newmark-β method

Reviewers' comments:

1. Please explaing in detail why ϕ3 and ϕ6 set zero (ϕ_3_=ϕ_3_=0)?. And in this paragraph: “Here, α_3_ = α_6_ = 0; at this node, the remaining 3 positions 2, 4, and 6 need to be investigated: 𝜶𝒋 = [𝜶_𝟐_, 𝜶_𝟒_, 𝜶_𝟔_]𝑻 where j = 2, 4, 6”. Author set ϕ6 = 0, but the ϕ6 are investigted. Please explaining more this problem?
2. Retype the symbol of “Poisson coefficient” in Table 1.
3. In this studay, Did author used Implicit or Explicit Newmark-β method?
4. In the investigation of “The Effect of Unbalance on Shaft Displacement”, results of displacement on the X-axis and Y-axis were obtained from Experimental Equipment or from numerial analysis? Why don’t show the comparison of Simulation and Experiment results in this investigation.
5. This study conclued that “These research results demonstrate that Newmark - β method used to analyzing the behavior of rotor systems with the reliability and high stability, fast convergence time and accuracy to 5%.”. Where did this conclusion show in paper?
