# Supplementary material for: Investigation on the influence of unbalanced shaft component in gearbox on displacement using the Newmark-β method
Source: PLoS One. 2025 Nov 5;20(11):e0335632. doi: 10.1371/journal.pone.0335632 (PMC12588525; doi:10.1371/journal.pone.0335632)
Supplement: Attachment — Submitted filename: Response to Reviewers.docx [file pone.0335632.s004.docx]

Effect of unbalanced parameters on power transmission shaft displacement in gearbox system using the Newmark-β method

Dear Editor and Reviewer:
The revised paper is submitted. The corrections made in the revised paper are colored in red. Much appreciate your comments and help in the preparation of the paper.
**List of revisions in response to the comments from reviewer #1**

1. Please explaing in detail why ϕ_3_ and ϕ_6_ set zero (ϕ_3_=ϕ_6_=0)?. And in this paragraph: “Here, α_3_ = α_6_ = 0; at this node, the remaining 3 positions 2, 4, and 6 need to be investigated: 𝜶_𝒋_ = [𝜶_𝟐_, 𝜶_𝟒_, 𝜶_𝟔_] 𝑻 where j = 2, 4, 6”. Author set ϕ_6_ = 0, but the ϕ_6_ are investigted. Please explaining more this problem?

We rewrote the paragraph to explain the problem more clearly due to spelling errors

The displacement vector of the shaft node is:

$q_{i}=\left[ x_{i},y_{i},\alpha_{i} \right]^{T}$	(1)

## where the displacements along the X- and Y-axes and torsional angle α at the investigated positions are:

$x_{i}=\left[ x_{1},x_{2},x_{3},x_{4},x_{5},x_{6},x_{7} \right]^{T}$*;* $y_{i}=\left[ y_{1},y_{2},y_{3},y_{4},y_{5},y_{6},y_{7} \right]^{T}$

$\alpha_{i}=\left[ \alpha_{1},\alpha_{2},\alpha_{3},\alpha_{4},\alpha_{5},\alpha_{6},\alpha_{7} \right]^{T}$ where *i* is from 1 to 7;

and $\left\{ \begin{aligned} x_{i}=x_{n} or x_{i}=x_{m}+e_{m}cos\varphi_{m} \\ y_{i}=y_{n} or y_{i}=y_{m}+e_{m}cos\varphi_{m} \end{aligned} \right. , n=1, 3, 5, 7 ; m=2, 4, 6$

## Regarding the rotation angle, this study focuses on the shaft segment between nodes 2 and 6. Within this segment, the torsional angles at nodes 3 and 5 are assumed to be negligibly small: α_3_ = α_5_ = 0. As a result, the nodes requiring further consideration are nodes 2, 4, and 6: $\boldsymbol{\alpha}_{\boldsymbol{i}}\boldsymbol{=}\left[ \boldsymbol{\alpha}_{\boldsymbol{2}}\boldsymbol{,}\boldsymbol{\alpha}_{\boldsymbol{4}}\boldsymbol{,}\boldsymbol{\alpha}_{\boldsymbol{6}} \right]^{\boldsymbol{T}}$ where i = 2, 4, 6. The governing equation for the rotation angle is given as:

$\emptyset_{i}(t)=\alpha_{i}+\omega_{i}t+\emptyset_{0_{i}}$	(2)

where $\emptyset_{i}$*(t)* is the total rotation angle at node i as time t; $\emptyset_{0_{i}}$ is initial phase angle at node i; $\alpha_{i}$: torsional angle at node i; $\omega_{i}$: angular velocity at node i with i = 2, 4, 6;

The initial phase angle parameters have been updated in Table 1.

| $\emptyset_{0_{2}}$ | Initial phase deviation angle | 0 | rad |
| --- | --- | --- | --- |
| $\emptyset_{0_{6}}$ | Initial phase deviation angle | 0 | rad |

2. Retype the symbol of “Poisson coefficient” in Table 1.

We have rewritten the symbol “*υ”* in Table 1

3. In this study, Did author used Implicit or Explicit Newmark-β method?

Thank you for your comments. We have updated the manuscript to clarify.

In this study, the implicit Newmark–β method was employed, as it does not require adherence to the time step condition: $\Delta t\leq\frac{2}{\omega_{max}}=\frac{2}{319}=0.006$. The parameters $\gamma\mathrm{and}\beta$ were adjusted during the time-stepping loop and set to $\gamma=\frac{1}{2} \mathrm{and}\beta=\frac{1}{4}.$ A constant time step of Δt = 0.01 was selected for the simulations to ensure stability and computational efficiency [20].

4. In the investigation of “The Effect of Unbalance on Shaft Displacement”, results of displacement on the X-axis and Y-axis were obtained from Experimental Equipment or from numerial analysis? Why don’t show the comparison of Simulation and Experiment results in this investigation.

During the study, shaft displacement results along the X-axis and Y-axis were obtained using both numerical analysis and experimental measurements. A comparison between the simulation and experimental results is presented in Fig 8 and Table 2.

**Fig 8. X-axis displacement at node 4 between the Newmark–β numerical simulation**

**and experimental results at n = 2000 rpm.**

**Table 2. Measurement results of displacement in the X and Y axes at node 4**

| **Frequency (Hz)** | **Speed**  **(rpm)** | **Newmark - β method** | | **Experimental data** | | **Relative Error** | |
| --- | --- | --- | --- | --- | --- | --- | --- |
|  |  | **X (mm)** | **Y (mm)** | **X (mm)** | **Y (mm)** | **X** | **Y** |
| 34 | 2000 | ± 0.043 | ± 0.071 | ± 0.046 | ± 0.081 | 6 % | 12 % |
| 51 | 3000 | ± 0.095 | ± 0.108 | ± 0.101 | ± 0.119 | 15 % | 18 % |

5.This study conclued that “These research results demonstrate that Newmark - β method used to analyzing the behavior of rotor systems with the reliability and high stability, fast convergence time and accuracy to 5%.”. Where did this conclusion show in paper?

This conclusion is summarized in Table 2, and we have updated the manuscript to present it more clearly.

**Table 2. Measurement results of displacement in the X and Y axes at node 4**

| **Frequency** (Hz) | **Speed**  (rpm) | **Newmark - β method** | | **Experimental data** | | **Relative Error** | |
| --- | --- | --- | --- | --- | --- | --- | --- |
|  |  | **X (**mm**)** | **Y (**mm**)** | **X (**mm**)** | **Y (**mm**)** | **X** | **Y** |
| 34 | 2000 | ± 0.043 | ± 0.071 | ± 0.046 | ± 0.081 | 6 % | 12 % |
| 51 | 3000 | ± 0.095 | ± 0.108 | ± 0.101 | ± 0.119 | 15% | 18 % |

The relative error in the X-axis displacement at node 4, at a rotation speed of 2000 rpm, is calculated as follows:

$$\varepsilon=\frac{0.046-0.043}{0.046}=6 \%$$

## The result show that when the rotor operates below the first critical speed, the oscillations remain stable, with displacement amplitudes of X = ± 0.046 and Y = ± 0.081. A significant amplification in vibrational amplitude is observed as the rotor reaches the first critical speed, with displacements increasing to X= ± 0.101  and Y= ± 0.119 mm, clearly indicating resonance effects.

A comparison between the numerical results obtained using the Newmark–β method and the experimental data reveals a deviation of approximately 6%, which is considered acceptable for engineering analysis and validates the accuracy of the computational model.

**List of revisions in response to the comments from reviewer #2**

1. Your English is not good. Authors should use the Professional English Editing Service to improve your writing. In the submission of revision, authors must submit the certificate of this service.

We appreciate your suggestion. In response, we have thoroughly revised the manuscript to correct grammatical errors and have rewritten several sections to improve clarity and better convey our ideas.

2. Keyword section contain at least five key words

We added two keywords: dynamic modeling and rotor-bearing system.
3. Authors should use some potential tools to check AI in order to ensure the free-AI writing

Thank you for your comment. We have carefully reviewed the manuscript using AI-detection tools to ensure that the content is original and not generated by AI.

4. In each figure, components must be tagged by numbers or characters. Then, in the body text, authors must explain these components in details.

Thank you for your comment. We have revised all figures accordingly. Each component in the figures is now clearly labeled using numbers/characters, and detailed explanations of these components have been added in the corresponding sections of the manuscript.

1. *Rotating shaft – 2. Disk 1 – 3. Other disks*

**Fig 2. Schematic diagram of the rotor–bearing system.**

5. Each mathematical symbols in figures must be described in table or text

Thank you for your constructive comment. We have updated the manuscript to ensure that all mathematical symbols appearing in the figures are now clearly explained, either within the main text or in accompanying tables, for better clarity and understanding.

6. Remember that in the multiple images of one figure, each one must be labelled by (a), (b) or (c). Later, in the caption of figure, the description of these images should be denoted

Thank you for your comment. We have edited all images in the manuscript accordingly.

| *a) Shaft element model with two disks* | *b)* *Cross-section showing*  *eccentric mass location* |
| --- | --- |
|  |  |

**Fig 3. Schematic of rotor-bearing system with eccentric masses**

7. Characters in image are too small, it is difficult for readers to understand

Thank you for your comment. We have improved the clarity and size of the characters in the image to enhance readability and ensure better visual quality for the readers.

8. Fig. 6 should be removed since there is no meaningful. Authors stated that they indicate the dimensions of transmission shaft, however only lengths of them are displayed

Thank you for your comment. We agree with your comment that Fig 6 does not provide meaningful information, as it only shows the length of the drive shaft without sufficient dimensional details. Therefore, we have removed Fig 6 as suggested and updated Fig 3 in the manuscript.

| *a) Shaft element model with two disks* | *b)* *Cross-section showing eccentric mass location* |
| --- | --- |

**Fig 3. Schematic of rotor-bearing system with eccentric masses**

9. What is relation between Fig. 6 and Table 1?

We have removed Fig 6 and updated Fig 3 to make Table 1 clearer.

10. It is hard to read the parameters in both x axis and y axis?

We have edited all the images in the manuscript.

11. In Fig. 12, what does it mean, how can readers understand

To improve the clarity of Fig. 12, which has been moved to Fig. 11, and to help readers better understand its meaning, we have added Table 4, which provides the detailed data corresponding to Fig. 11. We hope this addition enhances the readers’ comprehension and makes the figure more informative.

**Fig 11. Horizontal displacement of node 4 for m_trial_ = 20g at speeds of 800, 1500 and 2000 rpm.**

**Table 4. Displacement measurement results at node 4 under 20g test mass at 800, 1500, and 2000 rpm.**

| **No.** | **Speed** (rpm) | **m_trial_** (grams) | **Displacement at x_4_** (mm) |
| --- | --- | --- | --- |
| 1 | 800 | 20 | ± 0.055 |
| 2 | 1500 | 20 | ± 0.073 |
| 3 | 2000 | 20 | ± 0.098 |

## Fig 11 and Table 4 illustrates the variation in horizontal (X-axis) displacement at node 4 under different operating speeds. The results clearly show an increasing trend in displacement amplitude as the rotational speed increases, particularly as the rotor approaches its first critical speed. This behavior reflects a corresponding rise in dynamic excitation, which is characteristic of resonance phenomena in rotor-dynamic systems.

12. Please improve the quality of all figures in this manuscript

Thank you for your helpful suggestion. We have improved the quality of all figures in the manuscript by enhancing their resolution, clarity, and overall visual presentation to ensure better readability and consistency.

13. I think that authors did not prepare this manuscript carefully

Thank you for your candid feedback. We sincerely regret any lack of clarity or oversight in the original submission. Based on your valuable comments, we have carefully reviewed and thoroughly revised the manuscript to improve its overall quality, including the figures, formatting, and technical descriptions. We truly appreciate your input, which has helped us enhance the manuscript significantly.

**List of revisions in response to the comments from reviewer #3**

1. Abstract must be re-written so that it reflects your works with significant contributions, briefly describe your method and insists on what technique you did

We have rewritten the Abstract

This study introduces an enhanced numerical approach for analyzing the dynamic behavior of a rotor-bearing system subjected to unbalanced excitation from a gearbox drive shaft. The Newmark-β integration method with the integration of a variable time-step algorithm was used, thus allowing to rapidly and accurately solve the system's nonlinear equations of motion without compromising stability. This technique enables a precise computation of displacement and torsional deformation of the rotating shaft during its operational cycle. The proposed computational model is validated against experimental data, showing deviations of less than 6%. A comprehensive parametric analysis is conducted to evaluate the influence of rotational speed, trial mass, and initial phase angle on the system dynamics. The findings confirm that our enhanced numerical approach yields rapid convergence and reliable predictions, making it a valuable tool for dynamic analysis of rotor-bearing systems.

2. Title is so boring, authors should use native English speaker to modify some errors in grammar and phrase

We have rewritten the Title:

Investigation on the Influence of Unbalanced Shaft Component in Gearbox on Displacement Using the Newmark-β Method

3. Reference section is very poor, commonly there are necessary to cite 30-40 publications in related fields. Hence you must invest more efforts to read additional papers. Some key developments such as 10.1177/1077546320953733, 10.1007/s11071-022-08100-3, 10.1016/j.ymssp.2023.110941 and 10.1016/j.ymssp.2022.109691 should be mentioned and analyzed in detail.

Thank you for your comment. The literature survey is updated in the revised version and we also rewrite the introduction section to clarify our idea in this work. We updated the manuscript by adding the reference list and also rewrote the introduction section.

4. Cited style should be changed. Term "author et al" is repeated so many times and should be replaced by the others

We have replaced the phrase "author et al" with others in the manuscript.

5. In the end of Introduction section, the contributions of this work must be declared

We have rewritten this section in the manuscript.

**This study represents a computational dynamic model for a rotor-bearing system under unbalanced excitation, using the Newmark-β method with a variable time-step algorithm. The approach will accurately predicts drive shaft displacement during operation. The key parameters, including rotational speed, trial mass, and initial phase angle, are put into account to evaluate their effects on system response.**

6. Also, the structure of this manuscript should be mentioned shortly at the end of introduction

Thank you for your comment. We have updated the manuscript.

7. All equations must be tagged in the order

Thank you for your feedback. We have thoroughly reviewed the manuscript and ensured that all equations are now correctly numbered in sequential order.
8. Fig. 1 is very blurred and authors must mention each component of this figure in the body text

Thank you for your helpful comments. We have updated Fig 1 for improved clarity and revised the manuscript to clearly describe each component, ensuring that readers can easily understand the system layout.

**Fig 1. Gear shaft assembly in the lathe gearbox.**

## Fig 1 shows shaft assembly in the lathe gearbox, comprising the drive shaft (3), bearings (1 and 5), and gears (2 and 4).

9. Fig. 2 is very basic and there is no need to display in the scientific research

Thank you for your comment. Although Fig 2 is simplified, it is essential for clarifying the system structure and notation used in the mathematical model. We have revised the Fig 2 and ensured all elements are clearly explained

2. *Rotating shaft – 2. Disk 1 – 3. Other disks*

**Fig 2. Schematic diagram of the rotor–bearing system.**

10. In one figure, if there are more than two images, authors must tag each individually and explain them in the caption of figure

Thank you for your comment. We have edited the figures in the manuscript.

| *a) Shaft element model with two disks* | *b)* *Cross-section showing eccentric mass location* |
| --- | --- |

**Fig 3. Schematic of rotor-bearing system with eccentric masses**

| *a) Model 3D* |  *b) Measurement setup of the rotating shaft* |
| --- | --- |

*1. Machine Pedestal – 2. Optical Sensor – 3. Acceleration Sensor – 4. Rotating shaft 5.Motor – 6. Encoder –7. Measurement sensor LK-G35 – 8.DAQ – 9.Computer*

**Fig 6. A vibration testing machine of rotating shaft.**

11. All mathematical symbols must be explained in detail such as x1, x2 ....

Thank you for your comment. We have carefully reviewed the manuscript and revised the relevant sections to ensure that all mathematical symbols are clearly defined and explained at their first appearance.

12. Fig. 4 lacks of the interactive forces/moments so that our readers could analyze your system

Thank you for valuable comment. Fig 4 has been revised to include the relevant interactive forces and moments, offering a clearer view of the system's dynamic behavior.

**Fig 4. Modelling of the study shaft segment from nodes 2, 4 and 6.**

13. Where is equation (8) derived ? which theory or theorem. Why vector of F force appear in the explanation but there is no vector in the computational equation

We have rewritten this paragraph in the manuscript.

## The Lagrange's equations of the second kind can be written in terms of the system as follows [19]:

$L=T-V$	(8)

$$\frac{d}{dt}\left( \frac{\partial T}{\partial\left\{ \dot{q}_{i} \right\}} \right)-\frac{\partial T}{\partial\left\{ \dot{q}_{i} \right\}}+\frac{\partial D}{\partial\left\{ \dot{q}_{i} \right\}}+\frac{\partial V}{\partial\left\{ \dot{q}_{i} \right\}}=F_{i}$$

## where T is the total kinetic energy, V is the total potential energy of the system; $\boldsymbol{F}_{\boldsymbol{i}}\boldsymbol{=}\boldsymbol{F}_{\boldsymbol{g}_{\boldsymbol{i}}}\boldsymbol{+}\boldsymbol{F}_{\boldsymbol{u}_{\boldsymbol{i}}}\boldsymbol{;}\boldsymbol{F}_{\boldsymbol{g}_{\boldsymbol{i}}}$ is force due to the mass of the node mass; $\boldsymbol{F}_{\boldsymbol{u}_{\boldsymbol{i}}}$ is centrifugal force due to unbalance.

14. In equation (3), what are these matrices and vectors? there is no explanation or explicit representation

Thank you for your comment. We have rewritten this paragraph in the manuscript.

## The kinetic energy of the system is expressed as follows:

$T=T_{t}+T_{r}=\frac{1}{2}\left\{ \dot{x} \right\}^{T}\left[ M_{c} \right]\left\{ \dot{x} \right\}+\frac{1}{2}\left\{ \dot{y} \right\}^{T}[M_{c}]\left\{ \dot{y} \right\}+\frac{1}{2}\left\{ \dot{\varphi} \right\}^{T}\left[ J_{c} \right]\left\{ \dot{\emptyset} \right\}$	(3)

where *T_t_* is translational kinetic energy; *T_r_*: rotational kinetic energy; $\left\{ \dot{x} \right\}$, $\left\{ \dot{y} \right\}$ are vectors of translational velocities in the X and Y – axes; $\left\{ \dot{\emptyset} \right\}$ is vector of angular velocities of the components; $\left[ M_{c} \right]$ is the mass matrix corresponding to the system from node 1 to node 7; *[*$J_{c}$*]* is the static moments of inertia concerning the study nodes 2, 4, 6.

15. It looks like that authors did not understand clearly what theoretical development was developed in this work

We acknowledge that language issues in the original manuscript may have obscured the theoretical contributions. The entire content of the manuscript has been carefully edited for clarity to better reflect the scope and originality of our work.

16. Why Table 5 is only validated in one mode such as speed 2000 rpm

In this study, rotor operation was examined over a wide speed range. It was found that at n_1_ = 800 rpm, n_2_ = 1500 rpm, and n_3_ = 2000 rpm—representing low, medium, and high speeds—the rotor demonstrated relatively stable dynamic behavior. These speeds were therefore selected for further analysis, with n_3_ = 2000 rpm maintained below 70% of the first critical speed (n_c_ = 3047 rpm) to ensure system stability, as referenced in [22]. Accordingly, in Table 5, only the speed of 2000 rpm (m_trial_ = 30g; position 0^0^-0^0^) was selected for comparison with the rotor operating under no-load conditions, as this speed shows the highest vibration amplitude according to Table 3.
