# Supplementary material for: Investigation on the influence of unbalanced shaft component in gearbox on displacement using the Newmark-β method
Source: PLoS One. 2025 Nov 5;20(11):e0335632. doi: 10.1371/journal.pone.0335632 (PMC12588525; doi:10.1371/journal.pone.0335632)
Supplement: Attachment — Submitted filename: Response to Reviewers_R2.docx [file pone.0335632.s005.docx]

**Investigation on the Influence of Unbalanced Shaft Component in Gearbox on Displacement Using the Newmark-β Method**

Dear Editor and Reviewers:
The revised paper is submitted. The corrections made in the revised paper are colored in red. Much appreciate your comments and help in the preparation of the paper.
**List of revisions in response to the comments from reviewer #2**

Reviewer #2:

1. *English is not good, authors should use the Professional English Editing Service to modify. In the submission of the revised manuscript, the certificate of this service should be submitted*

We sincerely appreciate the reviewer’s comment regarding the language quality. The authors have significantly revised the English text and explanation to the professional form.

2. *Table 1 which indicate the system parameters of simulations, must be proved the correctness and properness. Authors must cite the reference source where you get them*

We thank the reviewer for this important comment. Accordingly, the authors have added three references ([21], [22] and [23]) for the model parameters in the revised manuscript.

3. *Authors should produce a Table for abbreviation and symbol*

We would like to thank the reviewer for the valuable comment. In response, we have updated Table 1 in the revised manuscript to clarify the system parameters.

4. *Supporting information is very strange, authors must follow the instructions of this journal*

Thanks for comment, the supporting information has been re-formatted in the revised manuscript.

5. *The statement of Funding must contain the project code*

We highly appreciate the reviewer’s comment. The project code has been added to the funding statement in the revised manuscript.

6. *Conclusion section must contain future works. Authors must specify the potential developments for our readers*

We thank the reviewer for the helpful suggestion. We have included a brief discussion of potential future works in the conclusion section to highlight possible directions for further research.

7. *Ref no 19, 21, 22 must be replaced by recent works*

Thanks to the reviewer's comment, accordingly, references [19], [21] and [22] have been updated to [19], [24] and [25].

8. *Results are presented, but there is limited discussion comparing them with similar studies in the literature.*

We thank the reviewer for this valuable comment. In the revised manuscript, we have updated the Introduction section to provide a more comprehensive overview of previous studies and to emphasize that there are currently very few experimental works in this field. As a result, direct comparisons with existing studies are limited. Nevertheless, the experimental results presented in this work offer reliable data and can serve as a valuable reference for future research.
